# Supplementary material for: Comparing impacts of metal contamination on macroinvertebrate and fish assemblages in a northern Japanese river
Source: PeerJ. 2021 Jan 27;9:e10808. doi: 10.7717/peerj.10808 (PMC7847197; doi:10.7717/peerj.10808)
Supplement: Supplemental Information 1 [file peerj-09-10808-s001.docx]

Supplemental materials for

Comparing impacts of metal contamination on macroinvertebrate and fish assemblages in a northern Japanese river

Hiroki Namba, Yuichi Iwasaki, Kentaro Morita, Tagiru Ogino, Hiroyuki Mano, Naohide Shinohara, Tetsuo Yasutaka, Hiroyuki Matsuda, Masashi Kamo

Corresponding author: Yuichi Iwasaki, yuichiwsk@gmail.com

*Description of the field sampling performed in September 2018*

Field sampling similar to that reported in this study was performed on 26 and 27 September 2018. The report based on this sampling is written in Japanese and is publicly available at the website of the Ministry of Economy, Trade and Industry, Japan (URL: https://www.meti.go.jp/meti_lib/report/H30FY/itakuichiran-H30FY.pdf). The results are summarized in Table S1 and Figures S1–S3.

Benthic macroinvertebrates were collected from an area of 625 cm^2^ using a Surber net sampler (mesh size, 0.355 mm) at reference (R1–R3) and contaminated (S1a, S1b–S3) sites. At sites R4 and S4 we collected three replicate 625-cm^2^ samples for macroinvertebrates. The methods used for determining metal concentrations and for fish sampling are same as described in the main text.

Table S1. Water-quality parameters at study sites in the Tokushibetsu River system, northern Japan, in September 2018

| Site | Cu | Cd | Pb | Zn | CCU |
| --- | --- | --- | --- | --- | --- |
|  | Dissolved (µg/L) | | | |  |
| Contaminated sites |  |  |  |  |  |
| S1a | 1.5 | 0.26 | 0.65 | 42.1 | 12.4 |
| S1b | 1.6 | 0.30 | 0.62 | 42.2 | 13.1 |
| S2 | 0.9 | 0.22 | 0.07 | 26.7 | 7.1 |
| S3 | 0.6 | 0.08 | 0.04 | 11.3 | 3.1 |
| S4 | 0.4 | 0.01 | <0.005 | 7.2 | 0.9 |
| Reference sites |  |  |  |  |  |
| R1 | 0.1 | <0.005 | <0.005 | 0.6 | 0.1 |
| R2 | 0.1 | <0.005 | <0.005 | 2.3 | 0.2 |
| R3 | 0.1 | <0.005 | <0.005 | 4.8 | 0.4 |
| R4 | 0.1 | <0.005 | <0.005 | 1.6 | 0.3 |
| WQC | 1.3 | 0.05 | 0.19 | 16.8 |  |

CCU, cumulative criterion unit (see text for details); WQC, U.S. EPA chronic water-quality criterion at a water hardness of 10 mg/L (U.S. Environmental Protection Agency 2002). Limits of quantification for Cu, Zn, Cd, and Pb were 0.005, 0.1, 0.005, and 0.005 µg/L, respectively.


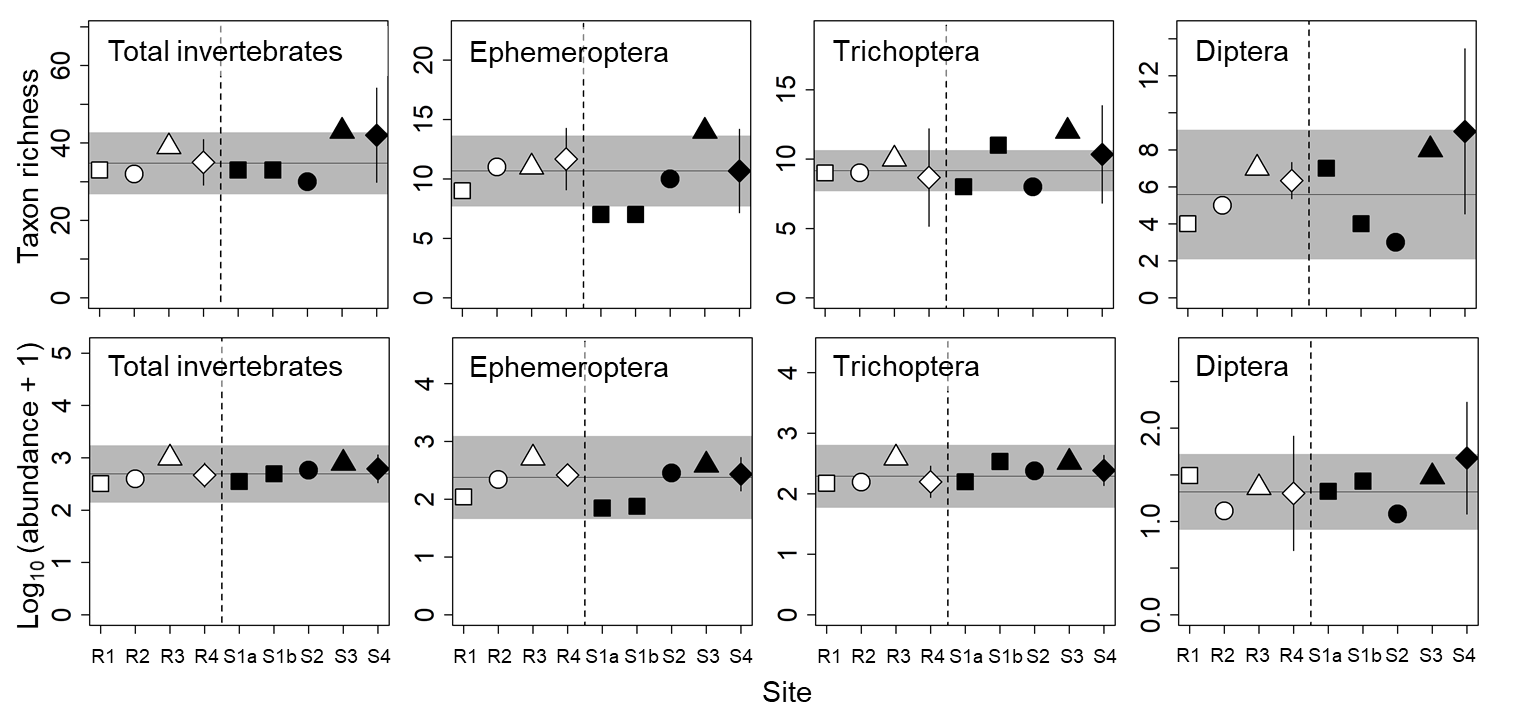
Figure S1. Abundance (number of individuals per 625 cm^2^) and taxon richness (number of taxa per 625 cm^2^) of macroinvertebrate communities at reference (R1–R4) and contaminated (S1a–S4) sites. The same symbols indicate sites with similar elevations. Error bars at R4 and S4 indicate 90% confidence intervals. Horizontal lines and gray areas indicate the means and 90% prediction intervals calculated from means for the four reference sites, respectively.


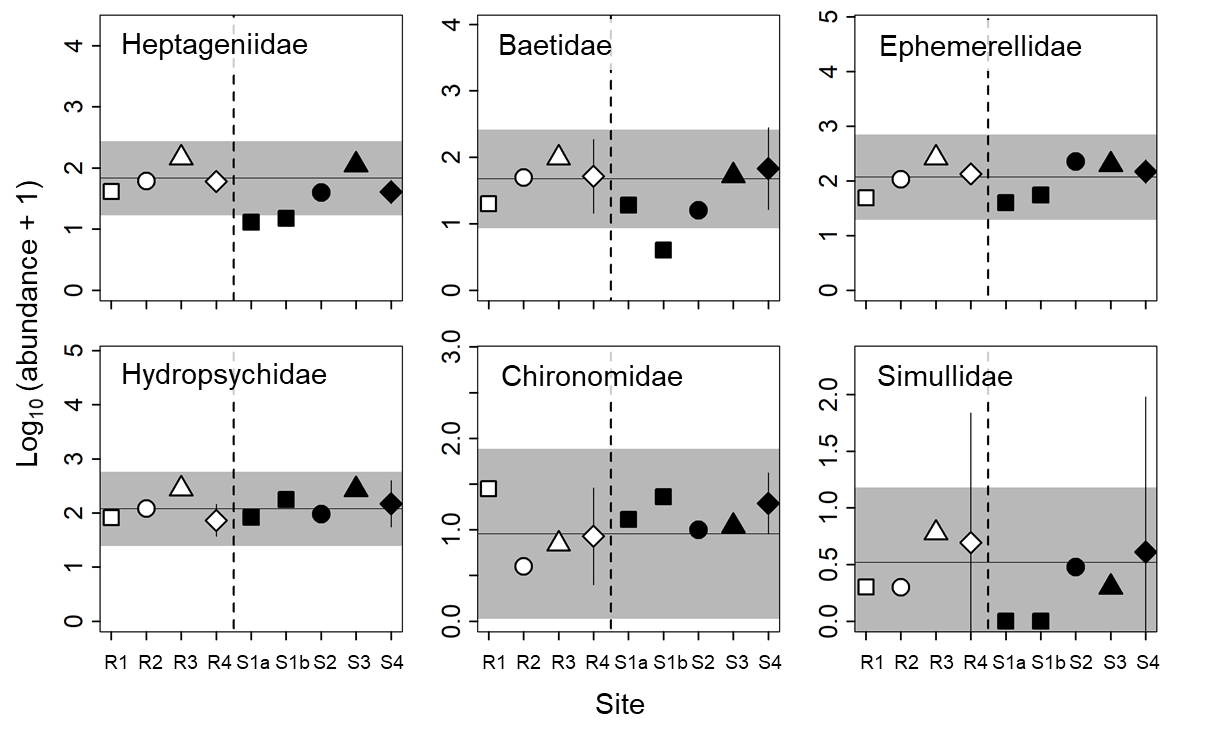


Figure S2. Abundance (number of individuals per 625 cm^2^) of dominant families of macroinvertebrates at reference (R1–R4) and contaminated sites (S1a–S4). The same symbols indicate sites with similar elevations. Error bars indicate 90% confidence intervals of site means (R4 and S4 only). Horizontal lines and gray areas indicate the means and 90% prediction intervals calculated from means for the four reference sites, respectively.


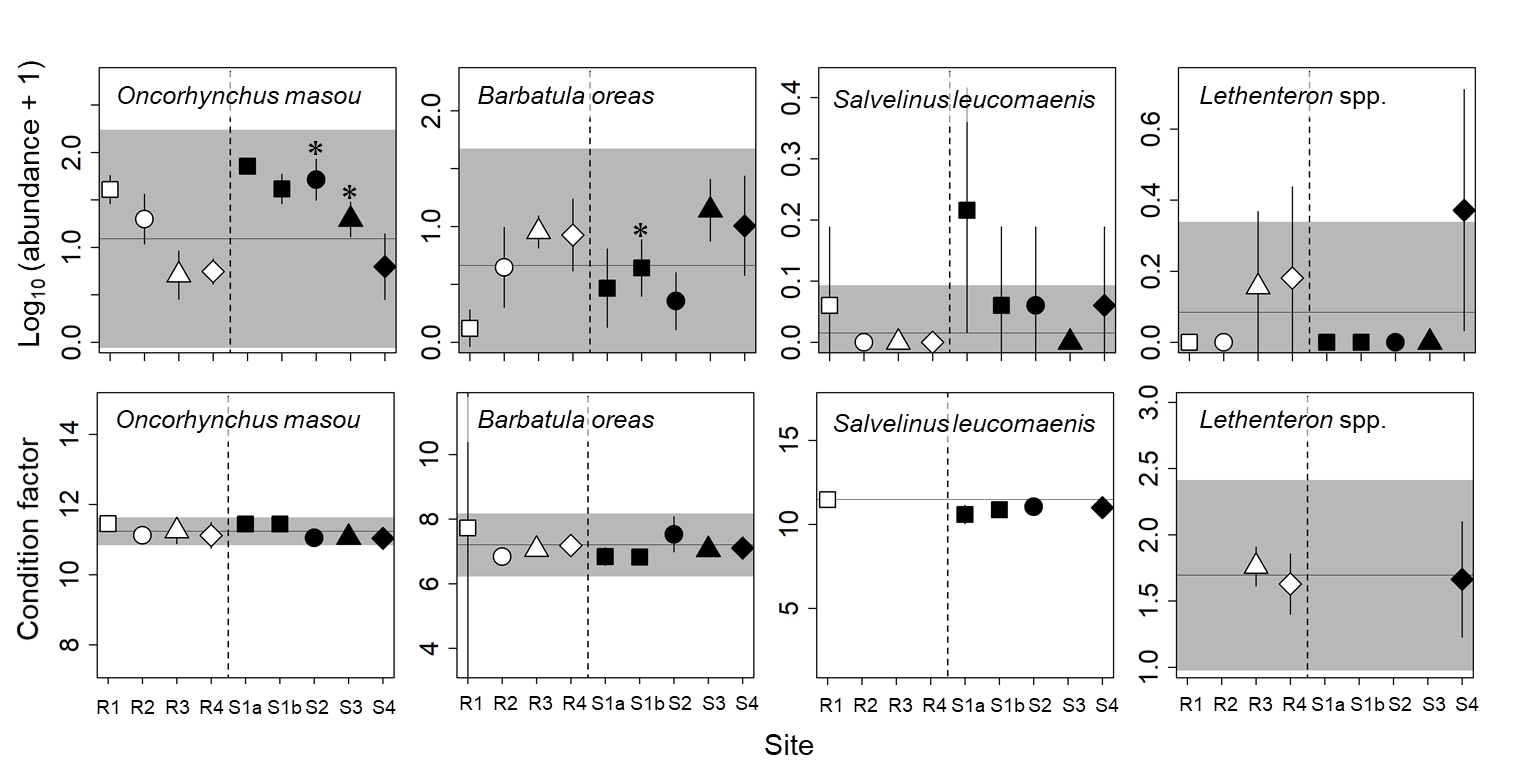
Figure S3. Abundance (number of individuals per 50 m^2^) and condition factor of fishes at reference (R1–R4) and contaminated (S1a–S4) sites. The same symbols indicate sites with similar elevations. Error bars indicate 90% confidence intervals. Horizontal lines and gray areas indicate the means and 90% prediction intervals calculated from means for the four reference sites, respectively. Asterisks indicate contaminated sites with values significantly lower or higher than the corresponding reference sites with similar elevations (*P* < 0.05).

**Reference**

U. S. Environmental Protection Agency 2002. National Recommended Water Quality Criteria: EPA822-R-02-047. Washington, DC.
